# *Badis kaladanensis*, a new fish species (Teleostei: Badidae) from Mizoram, northeast India

**DOI:** 10.1371/journal.pone.0246466

**Published:** 2021-07-28

**Authors:** Lal Ramliana, Samuel Lalronunga, Mahender Singh

**Affiliations:** 1 Department of Zoology, Pachhunga University College, Aizawl, Mizoram, India; 2 Department of Zoology, Mizoram University, Aizawl, Mizoram, India; 3 Molecular Biology & Biotechnology Division, ICAR-National Bureau of Fish Genetic Resources, Lucknow, Uttar Pradesh, India; Nanjing Agricultural University, CHINA

## Abstract

*Badis kaladanensis*, a new percoid fish is described from the Kaladan basin of Mizoram, northeast India. It belongs to the *Badis badis* species group but can be easily distinguished from its congeners, except from *B*. *kanabos* and *B*. *tuivaiei*, in having a dark blotch on the dorsal fin between the base of 3^rd^ to 5^th^ spines. It is further distinguished from *B*. *kanabos* in having more scales in lateral row (27–30 vs. 25–26), more circumpeduncular scale rows (18–20 vs. 16–17) and smaller eye (7.5–8.9% SL vs. 9.5–12.7); and from *B*. *tuivaiei* in having fewer vertebrae (28–29 vs. 30–31) and more rakers on the first gill arch (9 vs. 6–8). The analysis of the mitochondrial DNA (*coi* and *cytb*) revealed the distinctness of *B*. *kaladanensis* from all other *Badis* species with the interspecific distance ranges from 5.4–20.4%. (*coi*) and 5.1–26.3% (*cytb*).

## Introduction

Fishes of the genus *Badis* Bleeker 1854 are small freshwater fish normally inhabiting small streams or hill streams with slow to moderate flow, coastal drainages or ditches with stagnant waters. They are widely distributed over the Indian subcontinent, Pakistan, Nepal, Bangladesh, Myanmar, Peninsular Thailand, the Mae Khlong drainage, and part of the Mekong basin in South East Asia as well as the Upper Irrawaddy in southern Yunnan, China [1]. They belong to the family Badidae which consist of two genera viz., *Badis* and *Dario*. The genus *Badis* is distinguished from the genus *Dario* in having tubed lateral-line scales (vs. absent); 2–4 dentary foramina (vs. none); 3-toothed hypobranchial (vs. edentulous); short pelvic fin in males, not reaching the first anal-fin spine (vs. reaching beyond origin of anal fin); short dorsal fin lappets and rounded caudal fin (vs. prolonged and truncated respectively) [2].

Species belonging to the genus *Badis*, particularly the *B*. *badis* species group, exhibit very similar morphological traits including colour pattern [3]. Due to poor morphological diagnostic characters among some *Badis* species, accurate identification and/or delimitation of species remains a challenge. For instance, among the 25 currently recognized species of the genus *Badis* [4], based on published morphological data, *B*. *dibruensis* and *B*. *pancharatnaensis* cannot be distinguished from *B*. *badis*, and similarly, *B*. *triocellus* cannot be distinguished from *B*. *singenensis* [3]. The molecular approach of species delineation has been proved efficient and helpful when integrated with the conventional taxonomic approach [5–7]. It is therefore necessary to integrate morphological and the molecular approach for species delimitation, particularly among the *Badis* species.

An ichthyological survey conducted along the Palak River and Sala River, tributaries of the Kaladan River, resulted in the collection of a *Badis* species which differs from the already reported congeners. A comparison of the morphology and mitochondrial DNA (*coi* and *cytb*) sequences of the species with congeners revealed it to be an unnamed species, which is described herein as *Badis kaladanensis*, new species.

## Material and methods

The live fishes were collected specifically for this study after permission from Institutional Animal Ethics Committee of Pachhunga University College, Aizawl-Mizoram, India (approval no. PUC-IAEC-2011-A02). The study did not involve any endangered species or protected areas or private water bodies. The fishes were handled according to the guidelines laid down by the Committee for the Purpose of Control/ Supervision of Experiments on Animals (CPCSEA), and Animal Dissection Monitoring Committee of Pachhunga University College, Aizawl-Mizoram, India. In brief the live specimens of *Badis kaladanensis* sp.nov., were anesthetized using buffered solution of MS222 (TMS, Tricaine methanesulfonate) at the concentration of 70 mg/L in water, in a tank well oxygenated with external oxygen supply. The concentration of anesthesia was increased to 280 mg/L for euthanizing the fishes for preservation of voucher specimens. The fishes were kept for additional 20 minutes at this concentration after the opercular movement stopped in all fishes. In India no additional permission is required for collection and use of biodiversity within country.

**Fig 1:**
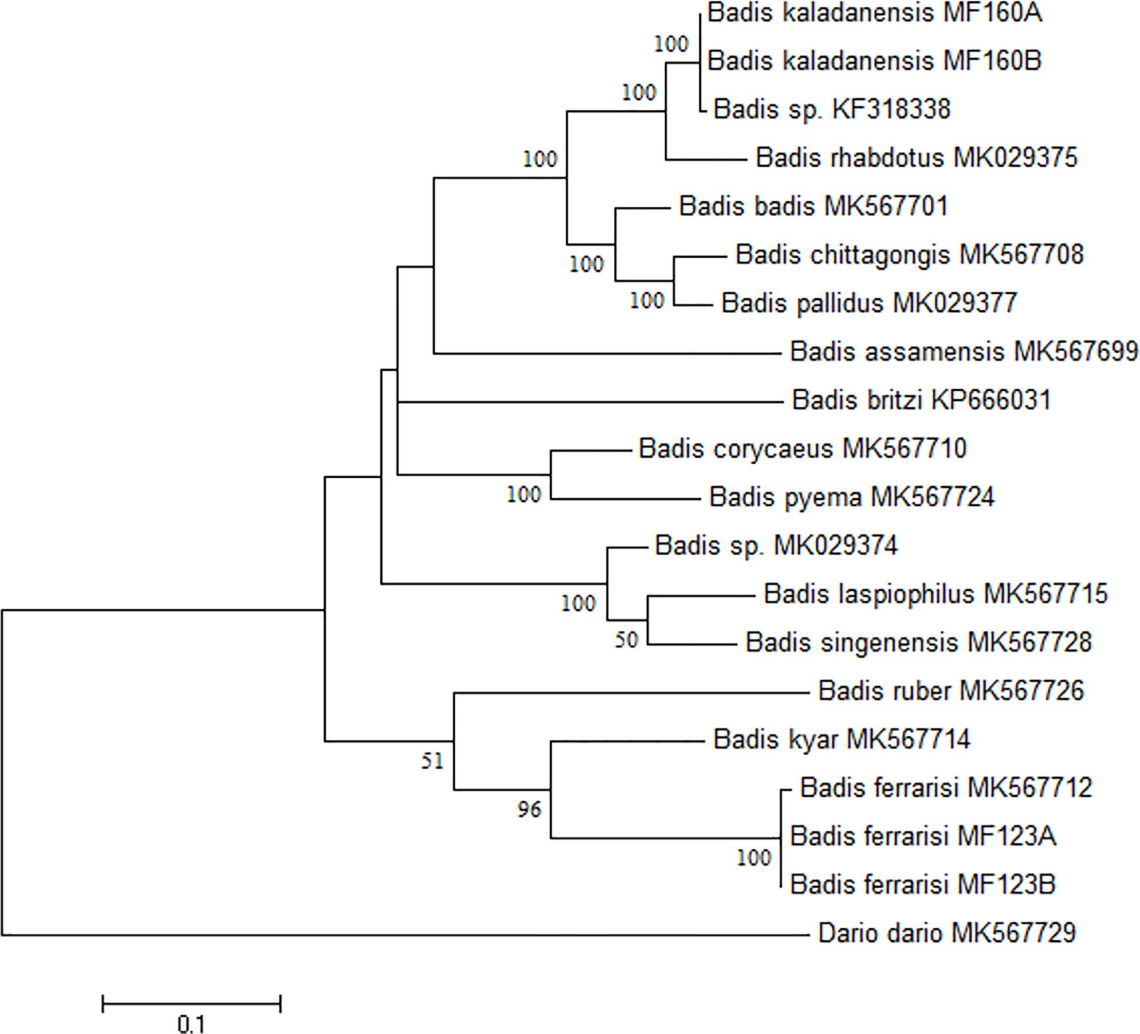
Maximum Likelihood (ML) tree of *coi* dataset of 32 sequences using HKY+G+I nucleotide substitution model with 1000 bootstraps.

A small amount of muscle was collected from the right side of each fish specimen and kept in 95% ethanol for DNA extraction. The fish specimens were fixed in 10% formalin and later transferred to 70% ethanol preservative. Counts and measurements were made on the left side of specimens, whenever possible, following Kullander & Britz [2]. Measurements were made point to point with digital calipers to the nearest 0.1 mm. Measurements of body parts are given as proportions of standard length (SL). For vertebral counts, four specimens were cleared and stained in alizarin. Vertebral count includes each of the first four vertebrae of the Weberian apparatus. Fin rays were counted using a stereomicroscope and were confirmed through cleared and stained specimens. The small posterior-most rays of the dorsal and anal fins, articulating with the same pterygiophore were counted together with the preceding ray as 1. Description and numbering of the laterosensory system on the head follows Fig 1 in Kullander & Britz [2]. Numbers in parentheses after a meristic value indicate the frequency of that value. All morphological measurements and meristic counts of *Badis kaladanensis* sp. nov. are given in Table 1.

**Table 1:** Biometric data for *Badis kaladanensis* (ZSI FF 5404, 5405; PUCMF 15001, 15002, 15003) (n = 19). Ranges include value of holotype.

	holotype	range	mean	SD
Standard length (mm)	48.6	28.2–49.3		
**In % standard length**				
Head length	29.2	28.1–30.9	29.3	0.7
Snout length	6.8	6.1–7.4	6.7	0.4
Orbital diameter	7.6	6.1–9.3	8.1	0.8
Interorbital width	4.5	4.2–6.0	5.1	0.6
Upper jaw length	9.3	5.9–9.9	8.8	1.0
Lower jaw length	11.1	9.6–12.2	11.3	0.6
Body depth	28.8	27.2–32.9	29.9	1.9
Pelvic fin length	25.1	23.2–27.2	24.8	0.9
Pelvic to anal fin distance	32.3	29.4–34.1	32.4	1.3
**In % head length**				
Snout length	23.2	20.7–25.2	22.9	1.3
Orbital diameter	26.1	20.8–31.5	27.7	2.5
Interorbital width	15.5	14.6–20.7	17.5	2.0

Type specimens were deposited at the Zoological Survey of India, Kolkata (ZSI) and Pachhunga University College Museum of Fishes, Mizoram (PUCMF). Other collection code used herein is MUMF for Manipur University Museum of Fishes.

### DNA extraction, PCR amplification and DNA sequencing

Genomic DNA was extracted from ~ 40 mg of tissue following Sambrook & Russel [8]. The mitochondrial genes *cytochrome oxidase subunit I* (*coi*) and *cytochrome b* (*cytb*) were selected for molecular analysis. Amplification was done using the primers: Fish-F1 (forward) and Fish-R1 (reverse) [9] for *coi* and L14724 (forward) and H15915 (reverse) [10] for *cytb* using Veriti 96 fast thermal cycler (Applied Biosystems, Inc., USA). A total of 25 μl PCR volume containing 1X buffer, 100 μM dNTPs, 2 mM MgCl_2_, 5 pmol of each primer, 2U Taq DNA polymerase and 100 ng template DNA were prepared. The PCR conditions (for both *coi* and *cytb*) are: initial denaturation of 3 min at 94°C, followed by 35 cycles of denaturation at 94°C for 50 sec, annealing at 54°C (49°C for *cytb*) for 30 sec., extension at 72°C for 80 sec. with final extension of 10 min at 72°C. Sequencing was performed in forward and reverse direction using an automated ABI 3500 Genetic Analyzer (Applied Biosystems, Inc, USA).

### DNA sequence analysis

The four sequences (2 each for *B*. *kaladanensis* and *B*. *ferrarisi*) obtained were analysed with the available GenBank sequences of *Badis* species (27 for *coi* and 31 for *cytb*) along with *Dario dario* as outgroup (KC774648 for *coi* and AY330958 for *cytb*) (Table 2).

**Table 2:** Collection localities/ sources, voucher ID and GenBank accession numbers for taxa included in this study for *coi* and *cytb* dataset.

Species	Accn. No. *coi*	Accn. No. *cytb*	Lat-long	Reference
*Badis assamensis*	MK567699	MK567734	27.63 N 95.39 E	Kullander *et al*., 2019 [24]
*Badis badis*	MK567701	MK567736	22.97 N 88.83 E	Kullander *et al*., 2019 [24]
*Badis blosyrus*	-	AY330941	Assam, India	Rüber *et al*., 2004 [22]
*Badis britzi*	KP666031	-	13.92 N 74.89 E	Dahanukar *et al*., 2015 [23]
*Badis chittagongis*	MK567708	MK579359	21.27 N 92.06 E	Kullander *et al*., 2019 [24]
*Badis corycaeus*	MK567710	MK567739	25.34 N 97.28 E	Kullander *et al*., 2019 [24]
*Badis ferrarisi*	MK567712	MK567741	24.18 N 94.37 E	Kullander *et al*., 2019 [24]
*Badis ferrarisi*	MW000670	MT997156	23.30 N 94.15 E	This study
*Badis ferrarisi*	MW000671	MT997157	23.30 N 94.15 E	This study
*Badis* cf. *kanabos*	-	MK579349	25.87 N 91.88 E	Kullander *et al*., 2019 [24]
*Badis kanabos*	-	AY330946	Assam, India	Rüber *et al*., 2004 [22]
*Badis* sp. Kolodyne	KF318338	-	22.07 N 92.92 E	Ramliana & Ghosh, 2017 (unpublished)
*Badis kaladanensis*	MW000668	MT997154	22.24 N 92.90 E	This study
*Badis kaladanensis*	MW000669	MT997155	22.20 N 92.89 E	This study
*Badis khwae*	-	AY330947	Kanchanaburi, Thailand	Rüber *et al*., 2004 [22]
*Badis kyar*	MK567714	MK567743	25.34 N 97.28 E	Kullander *et al*., 2019 [24]
*Badis laspiophilus*	MK567715	MK567744	India	Kullander *et al*., 2019 [24]
*Badis pallidus*	MK029377	MK579350	22.46 N 91.80 E	Kullander *et al*., 2019 [24]
*Badis pyema*	MK567724	MK567747	27.33 N 97.38 E	Kullander *et al*., 2019 [24]
*Badis rhabdotus*	MK029375	MK579364	Meghalaya India	Kullander *et al*., 2019 [24]
*Badis ruber*	MK567726	MK567749	17.39 N 95.91 E	Kullander *et al*., 2019 [24]
*Badis siamensis*	-	AY330955	Phuket, Thailand	Rüber *et al*., 2004 [22]
*Badis singenensis*	MK567728	MK567751	26.34 N 93.87 E	Kullander *et al*., 2019 [24]
*Badis* sp.	MK029374	MK579365	24.92 N 92.79 E	Kullander *et al*., 2019 [24]
*Badis* sp.	-	AY330957	Assam, India	Rüber *et al*., 2004 [22]
*Dario dario*	MK567729	MK567752	India	Kullander *et al*., 2019 [24]

**Fig 2:**
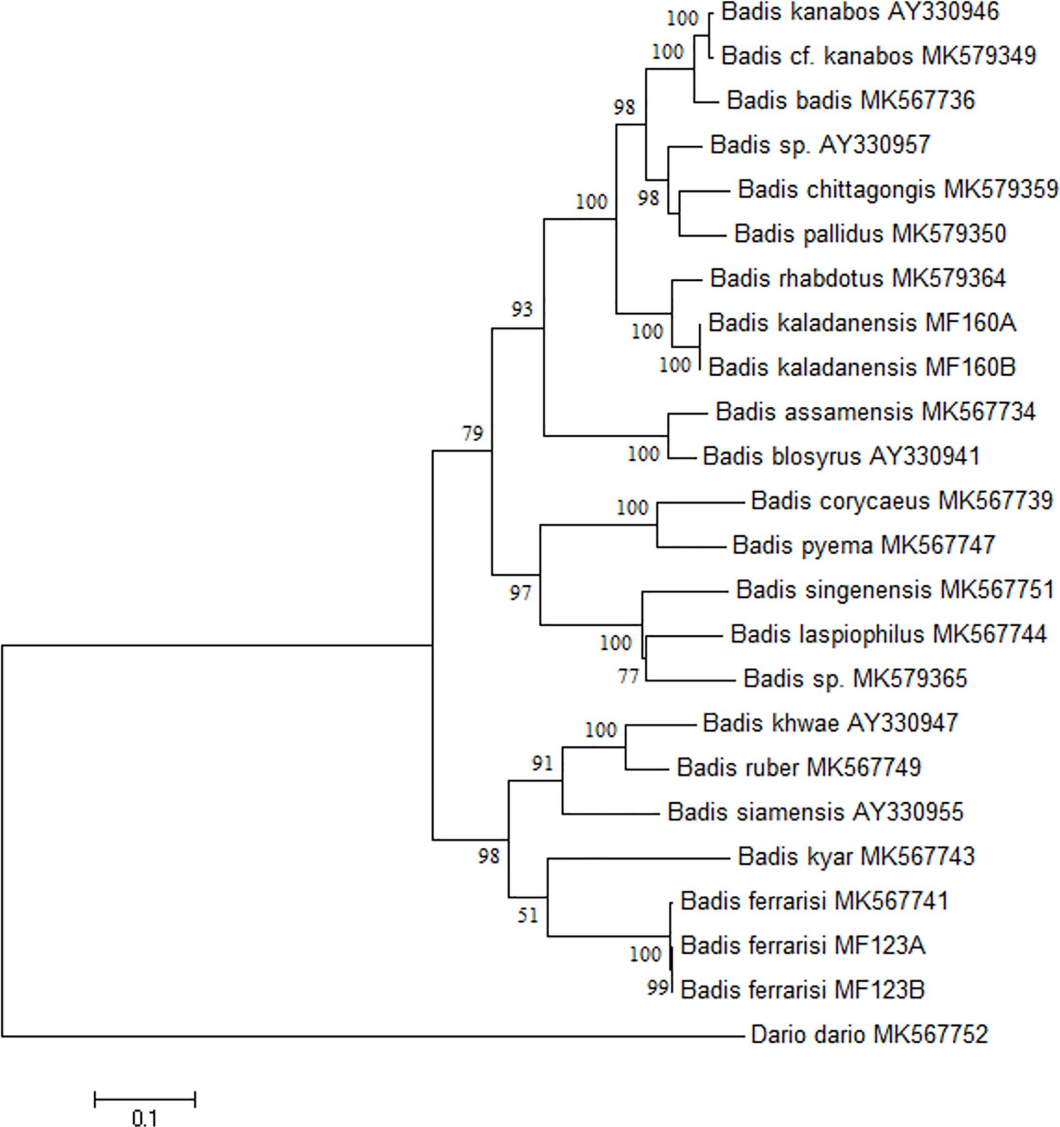
Maximum Likelihood (ML) tree of *cytb* dataset of 36 sequences using HKY+G+I nucleotide substitution model with 1000 bootstraps.

Sequences were aligned using CLUSTAL_W module integrated in MEGA 7 (Molecular Evolutionary Genetics Analysis) software [11]. The sequences were blasted in NCBI (http://www.ncbi.nlm.nih.gov) for the nearest matches and submitted to NCBI GenBank (Accn. Nos. MT997154-MT997157 for *cytb* and MW000668-MW000671 for *coi*). The genetic distance was calculated by averaging pairwise comparisons of sequences across close relatives of *Badis* in MEGA7. The maximum likelihood (ML) tree was constructed using *coi* (Fig 1) and *cytb* dataset (Fig 2). Based on the lowest BIC (Bayesian Information Criterion), the best fit nucleotide substitution model for both the *coi* and *cytb* dataset was HKY+G+I given by Hasegawa *et al*. [12].

### Nomenclature acts

The electronic edition of this article conforms to the requirements of the amended International Code of Zoological Nomenclature, and hence the new names contained herein are available under that Code from the electronic edition of this article. This published work and the nomenclatural acts it contains have been registered in ZooBank, the online registration system for the ICZN. The ZooBank LSIDs (Life Science Identifiers) can be resolved and the associated information viewed through any standard web browser by appending the LSID to the prefix "http://zoobank.org/". The LSID for this publication is: urn: lsid: zoobank.org:pub:9A23E022-F6F5-4226-BCD3-9F9D512AD4A8. The electronic edition of this work was published in a journal with an ISSN, and has been archived and is available from the following digital repositories: PubMed Central, LOCKSS.

## Results

### Molecular analysis

The *coi* and *cytb* gene sequences of *Badis kaladanensis* generated in this study are distinct compared to available GenBank sequences of other *Badis* species (5.4–20.4% for *coi* and 5.1 to 26.3 for *cyt b*), with the exception of one *coi* sequence of an unconfirmed *Badis* species, submitted earlier by first author (KF318338), with only 0.3% K2P distance, indicating that they are conspecific. *Badis kaladanensis* clustered in the same clade with species of the *Badis badis* species group [2], indicating that *B*. *kaladanensis* belongs to the *B*. *badis* species group.

### Taxonomy

#### *Badis kaladanensis* sp. Nov

(Fig 3).

**Fig 3:**
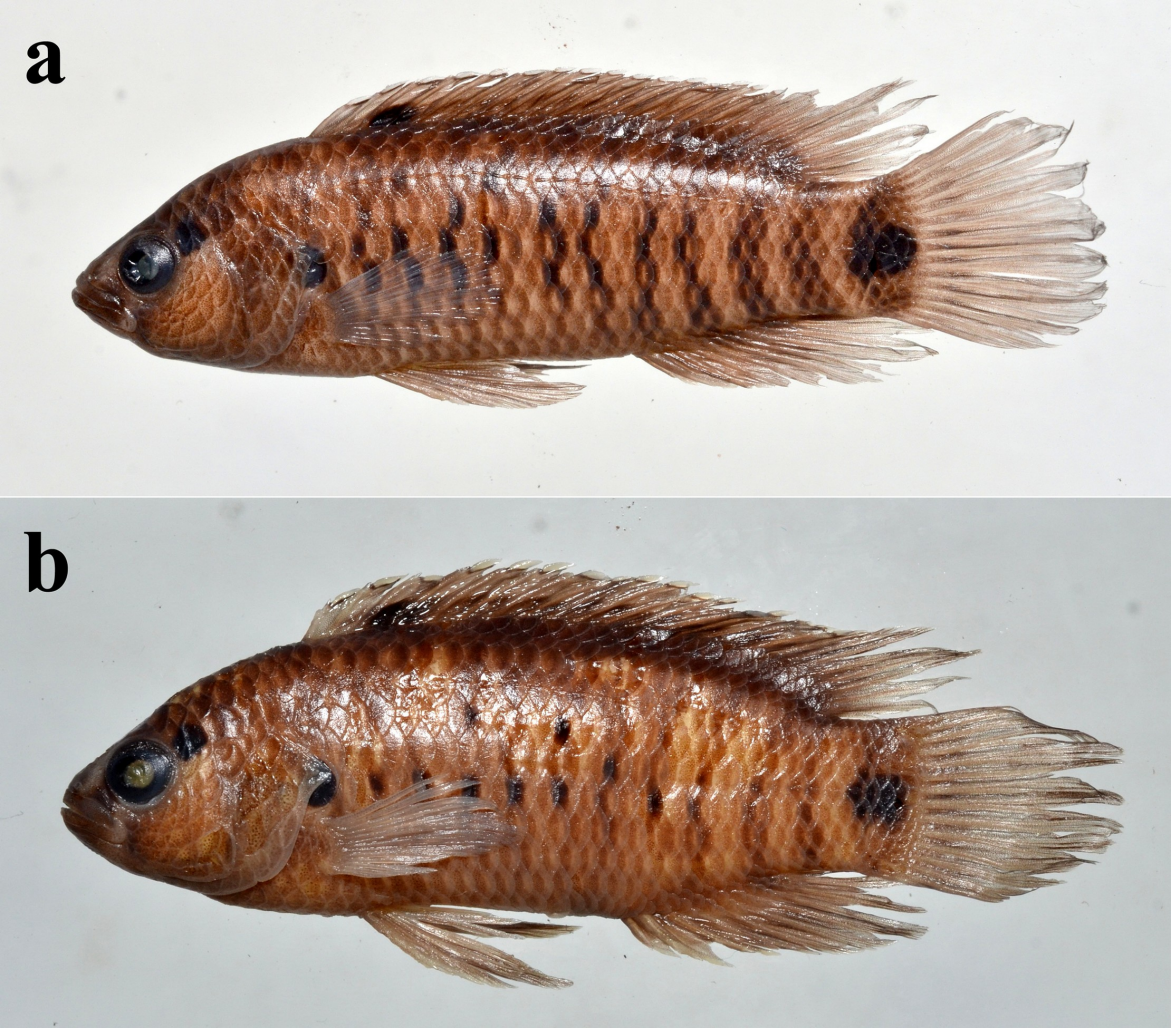
a) *Badis kaladanensis*, holotype, ZSI FF 5404, 48.6 mm SL; b) Paratype, PUCMF 15002, 45.2 mm SL.

urn:lsid:zoobank.org:act:4D86FCA2-EEEB-43A1-B20F-A0C2884D96D6

#### Holotype

ZSI FF 5404, 48.6 mm SL; India; Mizoram, Palak river in the vicinity of Phurra Village; 22°14’12"N; 92°53’59"E; Lalronunga *et al*., 15 Nov. 2012.

#### Paratypes

ZSI FF 5404 (2), 40.1–49.3 mm SL; data same as holotype. PUCMF 15001 (4), 28.2–44.8 mm SL; same data as holotype. PUCMF 15002 (5), 32.2–46.7 mm SL; India; Mizoram, Palak river in the vicinity of Palak Lake; 22°12’18"N; 92°53’33"E; Vanlalmalsawma & Lalramliana, 7 Dec. 2011. PUCMF 15003 (3), 35.3–47.5 mm SL; India; Mizoram, Sala river in the vicinity of Lungpuk Village 22°04’05"N 92°55’19"E; Lalronunga *et al*., 14 Nov. 2012. PUCMF 15004 (4), 30.2–42.7 mm SL (cleared and stained); data same as holotype.

#### Diagnosis

*Badis kaladanensis* is distinguished from its congeners in having the following combination of characters: a post nuchal hump, a conspicuous dark blotch covering superficial part of cleithrum above pectoral-fin base, a dark anterior dorsal-fin blotch between 3^rd^ to 5^th^ spines (consistently present in live and preserved specimens), 27–30 scales in lateral row, 18–20 circumpeduncular scale rows, 28–29 vertebrae, 9 rakers on the first gill arch and lacking a dark blotch on the dorsolateral aspect of caudal peduncle.

#### Description

See Table 1 for morphometric data and Fig 3 for appearance. Body elongate, compressed laterally. Predorsal profile rising approximately evenly from tip of snout to first dorsal-fin spine (a post-nuchal hump present). Snout triangular or slightly pointed in lateral aspect. Orbit situated in anterior half of head at level of mid-lateral axis of body or slightly below it, diameter smaller than ⅓ of head length. Mouth oblique, protrusible, lower jaw projecting slightly beyond upper jaw, maxilla reaching to ⅓ of orbit, lower jaw articulation below middle of orbit. Opercular spine slender, with a sharp tip. Palatine, vomer and parasphenoid toothed.

Dentary pores 4 (d1–d4), anguloarticular pores 2 (aa1–aa2), preopercular pores 6 (p1–p6), nasal pores 2 (n1–n2), supraorbital pores 4 (f1–f4), extrascapular pores 3 (ex1–ex 3), posttemporal pores 2 (po1–po2), coronalis pore 1 (cor), lachrymal pores 3 (l1–l3), infraorbital pores 4 (io1–io4). A row of free neuromasts extending across gap between posterior most lachrymal and anterior most infraorbital pores.

Scales ctenoid on sides, cycloid on top of head. Predorsal scales anterior to coronalis pore 4 (4) or 5 (15), posteriorly 7 (4), 8 (6) or 9 (9). Cheek and opercular scales ctenoid, cheek scales extending posteriorly from termination of gape or below middle of orbit. Scales on cheek 4 rows (17), rarely 3 (2). Opercle with 3 rows of scales, 1 row each on preopercle, subopercle and interopercle. Scales in lateral row with 27 (4), 28 (4), 29 (9) or 30 (2). Lateral line with tubed scales and divided into two segments. Upper lateral line begins at dorsal origin of operculum. Lower lateral line usually begins at one scale anterior to vertical through posterior end of anal fin. Lateral-line tubed scales (upper/lower) with 21/6 (1), 22/5 (3), 22/6 (4), 23/6 (6), 24/5 (3), 24/6 (2). Circumpeduncular scale rows 18 (5), 19 (2), 20 (12). Dorsal-fin scale cover up to 2–3 scales wide; anal-fin scales cover three scales wide. Scales in vertical row 1½ above, 7 below lateral line. Gill rakers on the first gill arch 9 (4), branchiostegal rays 6 (4). Vertebrae (abdominal + caudal = Total vertebrae) 15+13 = 28 (3) or 15+14 = 29 (1)

Dorsal fin (spines/soft rays) with 16/9 (7) or 17/9 (12), its origin vertically above pectoral-fin origin and ends at two scales beyond vertical through base of last anal-fin ray. Dorsal- and anal-fin lappets with rounded tips reaching to almost ⅓ of caudal fin. Anal fin (spines/soft rays) with 3/7 (5) or 3/8 (14), reaching to about ¼ of caudal fin. Principal caudal rays 14 (19), fin rounded. Pectoral fin rounded with 13 (17) or 14 (2) rays, extending about ½ distance to anal-fin origin. Pelvic fin pointed with 1 (19) spine and 5 (19) branched fin rays, inner branch of second soft ray longest, usually not reaching vent (reaching up to vent in one paratype), slightly shorter in small specimens.

#### Coloration in preservative

Body pale brownish with brown or black markings. Preorbital stripe dark brown, continued across chin. Postorbital stripe distinct with a black blotch close to orbit, and a faint blotch one scale posterior to that blotch. No supraorbital stripe. Suborbital stripe indistinct below middle of orbit. No dark blotch on opercle. Cleithral blotch present, prominent and black.

Body with five pairs of dark brown vertical bars (usually composed of dark spots at bases of scales). Three pairs between head and anal fin-origin, and two pairs posterior to anal-fin origin. Bar above cleithral blotch absent or indistinct. Dorsal bar terminations form darker blotches, not observed in some specimens.

Dorsal fin dark brown or blackish with contrasting narrow white margin. A dark blotch at base between 3^rd^ to 5^th^ dorsal-fin spines. Caudal-fin base with a dark or brownish blotch covering the last lateral-line scale on body; a vertical black bar across caudal peduncle, absorbing the caudal base blotch. Anal and pelvic fins dark brown with distal margin usually hyaline.

**Fig 4:**
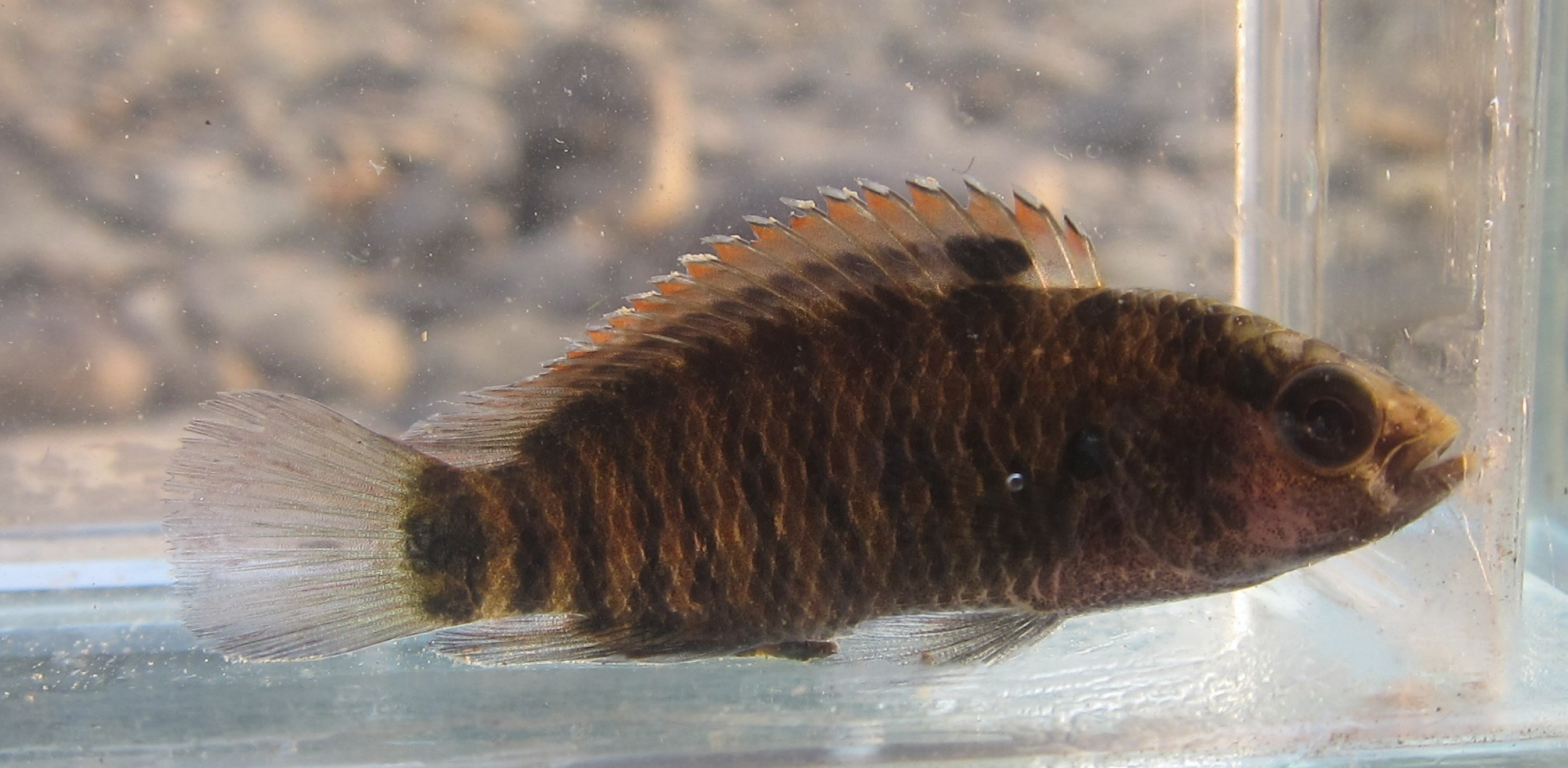
Live individual of *Badis kaladanensis*. PUCMF 15001, 44.8 mm SL.

**In life** (Fig 4).Similar to preserved specimens except that a dark band along middle of dorsal fin immediately distal to scaly basal cover, and sub-marginal part of dorsal fin orange.

#### Distribution and habitat

**Fig 5:**
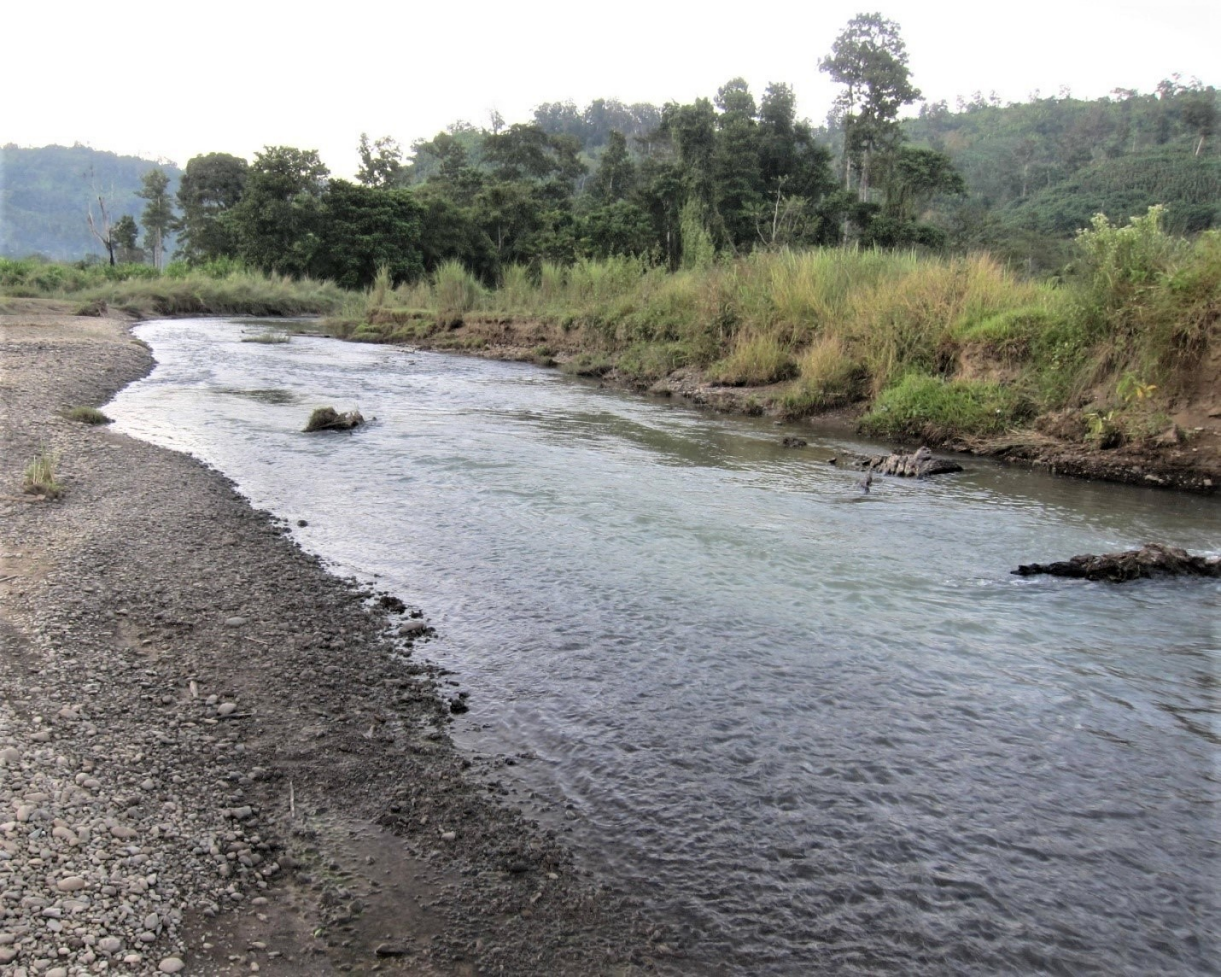
Type locality of *Badis kaladanensis* (Palak River, Mizoram, India).

**Fig 6:**
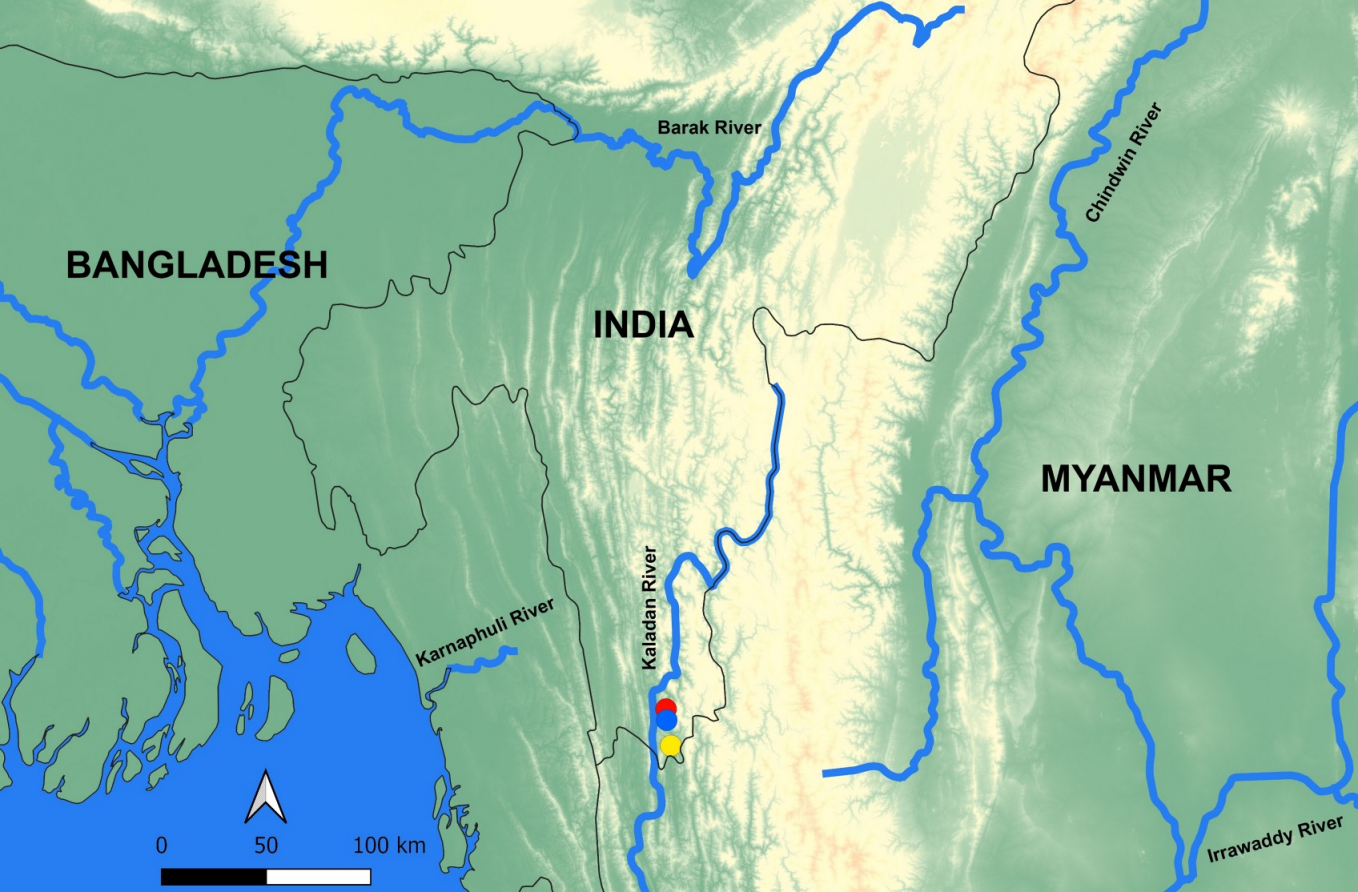
Map showing collection locality of *Badis kaladanensis* in the form of coloured dots. Red color dot represents Palak River in the vicinity of Phurra Village, Blue color dot represents Palak River in the vicinity of Palak Lake and yellow color dot represents Sala River in the vicinity of Lungpuk Village.

Known from the Palak River (Fig 5) and Sala River, a tributary of the Kaladan basin, in the vicinity of Phurra village and Lungpuk village respectively in Siaha District of Mizoram, India (Fig 6). It is found associated with *Olyra saginata*, *Pethia expletiforis*, *Rasbora rasbora* and *R*. *daniconius*.

#### Etymology

The species is named after the River drainage, the Kaladan River. An adjective.

## Discussion

*Badis kaladanensis* belongs to the *B*. *badis* group (cf. Kullander & Britz [2]), a group comprising *B*. *badis* (Hamilton), *B*. *kanabos* Kullander & Britz, *B*. *chittagongis* Kullander & Britz, *B*. *ferrarisi* Kullander & Britz [2] (Kullander & Britz 2002), *B*. *soraya* Valdesalici & van der Voort, *B*. *rhabdotus* Kullander *et al*., *B*. *pallidus* Kullander *et al*. [3] and both *B*. *dibruensis* and *B*. *tuivaiei* as they too possessed a conspicuous cleithral blotch, the synapomorphic character of the B. *badis* group stated by Kullander & Britz [2]. The previously included member of the group, *B*. *ferrarisi* [2], however, based on the genetic analysis, does not belong to this group and forms another group with *B*. *kyar* [3].

*Badis kaladanensis* markedly differs from members of the *B*. *badis* species group, apart from *B*. *kanabos* and *B*. *tuivaiei*, in having a dark blotch at base between 3^rd^ to 5^th^ dorsal fin spines (vs. absent in all other members). It differs from *B*. *kanabos* in having more scales in lateral row (27–30 vs. 25–26), more circumpeduncular scale rows (18–20 vs. 16–17), more vertebrae (28–29 vs. 26–28) and smaller eye diameter (7.5–8.9% SL vs. 9.5–12.7); from *B*. *tuivaiei* in having fewer vertebrae (28–29 vs. 30–31) and more rakers on the first gill arch (9 vs. 6–8).

The so far reported species of *Badis* from the Kaladan basin is *B*. *badis* [13] and the same species was reported form the Barak River drainage [14] inside Mizoram, India. Our previous collections from Tuirial and Tlawng Rivers (a tributaries of Barak River) and Tuichawng River (a tributary of Karnaphuli River) include *B*. *badis* and *B*. *rhabdotus*, but not a single specimen of *B*. *badis* from Kaladan basin. It is likely that the specimens previously reported as *Badis badis* from the Kaladan basin were in fact *B*. *kaladanensis*.

The morphological distinction of *Badis kaladanensis* from *B*. *badis*, *B*. *chittagongis*, *B*. *pallidus* and *B*. *rhabdotus* is less distinctive due to their overlapping characters. Moreover, *B*. *chittagongis* was described from the nearby basins, Karnaphuli and Matamohuri River drainage and *B*. *pallidus* from Karnaphuli and Sangu River drainage of Bangladesh. Since the mouth of Kaladan basin at Sittwe is very close to the mouth of the Karnaphuli, Matamohuri and Sangu River drainages at Bangladesh, *B*. *chittagongis* and *B*. *pallidus* are assumed to have wider distribution within Kaladan basin. However, we did not encounter any of those species from the basin during the study. *Badis kaladanensis* is a large species (attaining approximately 50 mm SL) compared to *B*. *badis*, *B*. *chittagongis*, *B*. *pallidus* and *B*. *rhabdotus* and possesses a post nuchal hump not observed in others. Besides, presence of dark blotch at base between 3^rd^ to 5^th^ dorsal-fin spines distinguished *B*. *kaladanensis* from *B*. *badis*, *B*. *chittagongis*, *B*. *pallidus* and *B*. *rhabdotus* (Table 3).

**Table 3:** Comparison of diagnostic characters among *Badis* congeners. ‘+’ indicates presence and ‘–’ indicates absence.

Serial number	Species	Cleithral blotch	Opercular blotch	Dark blotch at base between 3^rd^ to 5^th^ dorsal fin spines	Dorsolateral caudal blotch	Number of scales in lateral row	Circumpeduncular scales	Number of total vertebrae	Number of rakers on the first gill arch	Number of extrascapular pores
1	*B*. *badis*	+	+	–	–	25–28	19–20	26–28	5–9	2
2	*B*. *andrewraoi*	–	–	–	–	23–26	18	26	NA	2
3	*B*. *assamensis*	+	+	–	–	28–29	20	29–30	7–9	2
4	*B*. *autumnum*	–	+	–	–	25–27	16–18	26	NA	3
5	*B*. *blosyrus*	–	+	–	–	27–28	20	27–29	8–13	2
6	*B*. *britzi*	–	–	–	–	26–28	16	28	8	3
7	*B*. *chittagongis*	+	–	–	–	27–29	16–20	28–29	6–11	2
8	*B*. *corycaeus*	–	–	–	–	25–26	16	26–28	1–7	1
9	*B*. *dibruensis*	+	–	–	–	25–29	21–22	27	7	5
10	*B*. *ferrarisi*	+	+	–	–	25–26	18–20	26–28	6–7	1
11	*B*. *juergenschmidti*	–	–	–	–	25–26	NA	NA	NA	NA
12	*B*. *kaladanensis*	+	–	+	–	27–30	18–20	28–29	9	3
13	*B*. *kanabos*	+	+/–	+	–	25–26	16–17	26–28	6–9	2
14	*B*. *khwae*	+	–	+	+	27–28	16–18	27–28	4–8	2
15	*B*. *kyanos*	–	–	–	–	25–27	18–19	26	NA	4
16	*B*. *kyar*	–	–	+	–	26–27	16	27–29	5–9	2
17	*B*. *laspiophilus*	–	–	+	–	23–26	14–16	NA	NA	2
18	*B*. *pallidus*	+	–	–	–	26–28	15–17	27–29	6–10	2
19	*B*. *pyema*	–	–	–	–	27–28	16	28–29	5–7	1
20	*B*. *rhabdotus*	+	–	–	–	27–28	18	28–29	6	2
21	*B*. *ruber*	+	–	+	+	26–28	18–20	26–29	5–10	2
22	*B*. *siamensis*	+	+	–	+	25–27	16	26–28	5–8	2
23	*B*. *singenensis*	–	+	+	–	29–32	19–20	28	NA	2
24	*B*. *soraya*	+	–	–	–	25–27	18–22	27	NA	2
25	*B*. *tuivaiei*	+	–	+	–	26–32	16–20	30–31	6–8	2

Moreover, the interspecies distance of the DNA sequence markedly distinguished *B*. *kaladanensis* from *B*. *badis*, *B*. *chittagongis*, *B*. *pallidus* and *B*. *rhabdotus* (10.3–13.8% for *B*. *badis*, 11.7–12.4% for *B*. *chittagongis*, 5.9–5.1% for *B*. *rhabdotus* and 11.3–13.8% for *B*. *pallidus*).

*Badis kaladanensis* is also compared with members of other species groups. It differs from *B*. *kyar* (*B*. *kyar* group) in having more scales in lateral row (27–30 vs. 26–27) and the pattern of caudal-fin marking (vertical black bar across caudal peduncle absorbing the caudal base blotch vs. caudal fin marking with an entire curved band in *B*. *kyar*). Schindler & Linke [15] described *Badis juergenschmidti* from a river in south eastern central Myanmar which may be placed in the *B*. *kyar* group; however, they refrained from doing so because of remarkable differences between the two species. *Badis kaladanensis* differs from *B*. *juergenschmidti* in having more scales in lateral row (27–30 vs. 25–26) and presence of a dark blotch at cleithrum (vs. absent). Furthermore, *Badis kaladanensis* is distinguished from *B*. *ferrarisi* (*B*. *kyar* group) in having complete vertical bars on flank (vs. bars reduced and restricted to center of body), 3 extrascapular (vs. 1), and absence (vs. present) of opercular blotch; from *B*. *corycaeus* and *B*. *pyema* (*B*. *corycaeus* group) in lacking (vs. present) ocellated caudal base marking; from *B*. *assamensis* and *B*. *blosyrus* (*B*. *assamensis* group) in lacking (vs. present) opercular blotch; from *B*. *ruber*, *B*. *siamensis* and *B*. *khwae* (*B*. *ruber* group) in absence (vs. presence) of a dark blotch on the dorsolateral aspect of caudal peduncle; from *B*. *autumnum*, *B*. *andrewraoi* and *B*. *kyanos* (*B*. *autumnum* group [20]) in having (vs. lacking) a dark blotch at base between 3^rd^ to 5^th^ dorsal-fin spines and a conspicuous blotch covering the superficial part of the cleithrum above the pectoral-fin base; from *B*. *laspiophilus* and *B*. *singenensis* (*B*. *singenensis* group [21]) in lacking (vs. present) black blotch on both the soft anal and dorsal fin; and from *B*. *britzi* in having (vs. lacking) dark blotch at cleithrum and caudal-fin base, and a higher number of circumpeduncular scale rows (18–20 vs. 16).

The mitochondrial gene (*coi* and *cytb*) analysis of *Badis kaladanensis* and comparison with other *Badis* sequences available in GenBank revealed that it is significantly different from all of them, except the *coi* sequence of *Badis* sp. Kolodyne (0.3% only, indicating that they are conspecific). *Badis* sp Kolodyne is a specimen from the Lungbun river of Mizoram which is draining into the lower stretch of Kaladan drainage. This suggested that *B*. *kaladanensis* may have a wider distribution along the Kaladan River and its tributaries. Furthermore, the maximum likelihood tree inferred from *coi* and *cytb* confirmed that *B*. *kaladanensis* belongs to the *Badis badis* group. *Badis kaladanensis* is more closely related to *B*. *rhabdotus*, which has adjacent geographical distribution (type locality of *B*. *rhabdotus* being Karnafuli Drainage), compared to other species of *Badis badis* group. However as mentioned in the result section *B*. *kaladanensis* and *B*. *rhabdotus* belong to two distinct species (interspecies distance ranges from 4.9–5.1%). Moreover, the position in the tree (Figs 1 and 2) and the wide genetic distance from the closely related species confirms that *B*. *kaladanensis* is distinct from all other *Badis* species.

Despite the less pronounced morphological diagnostic characters among species of the *Badis*, many species have been described using morphological analysis leading to confusion. It is suggested to integrate morphological approach with molecular analysis. The integration of morphological and molecular approach of species delimitation will strengthen and speed up the taxonomy of *Badis* in future.

### Comparative material & sources

*Badis assamensis*: MUMF Per-51–54, 4, 41.6–55.8 mm SL; India: Assam: Dibru River, Dibrugarh.

*B*. *badis*: MUMF Per-55–65, 11, 23.5–28.7 mm SL; India: Manipur: Barak River. PUCMF 1065, 2, 30.9–43.8 mm SL; India: Mizoram: Tlawng River (Barak Drainage), Khamrang.

*B*. *blosyrus*: MUMF Per-66-68, 3, 36.8–38.9 mm SL; India: Arunachal Pradesh: Teju District: Teju River.

*B*. *dibruensis*: MUMF Per-95, holotype, 39.1 mm SL; India: Assam: Dibru River (Brahmaputra drainage), Dibrugarh. MUMF Per-96–97, paratypes, 37.4–39.8 mm SL; India: Assam: Dibru River (Brahmaputra drainage), Dibrugarh. Additional data from Geetakumari & Vishwanath [16].

*B*. *ferrarisi*: MUMF Per-69–75, 7, 32.0–44.0 mm SL; India: Manipur: Lokchao River. PUCMF 15055, 3, 25.7–29.8 mm SL; Myanmar: Tahan, Tahan Market (presumably from the nearby Myittha River, a tributary of Chindwin River).

*B*. *kanabos*: MUMF Per-76–81, 6, 48.7–54.9 mm SL; India: Manipur: Barak River.

*B*. *tuivaiei*: MUMF 5124, holotype, 34.6 mm SL; India: Manipur: Churachandpur District: Tuivai River (Brahmaputra drainage). ZSI FF 4159, 1, 57.0 mm SL: India: Manipur: Tamenglong district: Irang iver, a tributary of Barak River. Additional data from Vishwanath & Shanta [17].

Published information used for comparison: Dahanukar *et al*. [18] for *B*. *britzi*; Geetakumari & Kadu [19] for *B*. *singenensis*; Kullander & Britz [2] for *B*. *blosyrus*, *B*. *corycaeus*, *B*. *ferrarisi*, *B*. *kanabos*, *B*. *khwae*, *B*. *kyar*, *B*. *pyema*, *B*. *ruber* & *B*. *siamensis*; Kullander & Britz [2] and Kullander *et al*. [3] for *B*. *chittagongis*; Schindler & Linke [15] for *B*. *juergenschmidti*; Valdesalici & van der Voort [20] for *B*. *autumnum*, *B*. *andrewraoi*, *B*. *kyanos* and *B*. *soraya*; Valdesalici & van der Voort [21] for *B*. *laspiophilus*.
